# A Review of Whey Protein-Based Bioactive Delivery Systems: Design, Fabrication, and Application

**DOI:** 10.3390/foods13152453

**Published:** 2024-08-02

**Authors:** Liming Jiang, Zhiheng Zhang, Chao Qiu, Jinsheng Wen

**Affiliations:** 1 School of Basic Medical Sciences, Health Science Center, Ningbo University, Ningbo 315832, China; 2 State Key Laboratory of Food Science and Resources, School of Food Science and Technology, Collaborative Innovation Center of Food Safety and Quality Control in Jiangsu Province, International Joint Laboratory on Food Safety, Jiangnan University, Wuxi 214122, China

**Keywords:** whey protein, low-soluble active molecules, delivery system, dietary ingredient, interaction

## Abstract

The efficacy of many edible bioactive agents is limited by their low water dispersibility and chemical instability in foods, as well as by their poor bioaccessibility, low absorption, and metabolism within the human gastrointestinal tract. Whey proteins are amphiphilic molecules that can be used to construct a variety of edible carrier systems that can improve the performance of bioactive ingredients. These carrier systems are being used by the food and biomedical industries to encapsulate, protect, and deliver a variety of bioactive agents. In this article, we begin by providing an overview of the molecular and functional characteristics of whey proteins, and then discuss their interactions with various kinds of bioactive agents. The ability of whey proteins to be used as building blocks to assemble different kinds of carrier systems is then discussed, including nanoparticles, hydrogels, oleogels, bigels, nanofibers, nanotubes, and nanoemulsions. Moreover, applications of these carrier systems are highlighted. Different kinds of whey protein-based carriers can be used to encapsulate, protect, and deliver bioactive agents. Each kind of carrier has its own characteristics, which make them suitable for different application needs in foods and other products. Previous studies suggest that whey protein-based carriers are particularly suitable for protecting chemically labile bioactive agents and for prolonging their release profiles. In the future, it is likely that the applications of whey protein-based carriers in the food and pharmaceutical fields will expand.

## 1. Introduction

**Figure 1:**
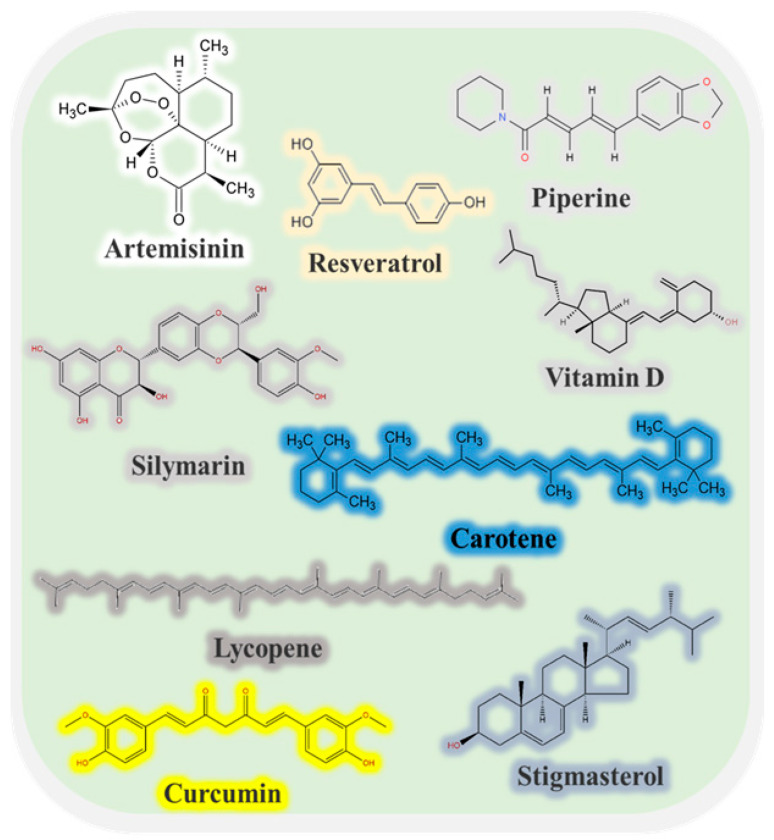
Some common low-water-soluble bioactive molecules and their chemical structures.

Many kinds of bioactive substance can be extracted from plants, animals, and microorganisms that may have beneficial effects on human health and wellbeing, including polyphenols, sterols, vitamins, minerals, omega-3 fatty acids, peptides, and oligosaccharides (Figure 1) [1,2,3]. For instance, these substances may exhibit anti-inflammatory, antimicrobial, antioxidant, antitumor, immunomodulatory, anti-atherosclerotic, and/or anti-aging activities [4,5]. However, there are still several challenges that need to be addressed before many of these bioactive substances can be successfully used as health-promoting ingredients in foods and medicines. First, some of them have poor solubility and wettability in water, which limits their introduction into aqueous-based products, such as beverages. Second, some of them are chemically labile and easily degrade when exposed to light, oxygen, or heat, thereby losing their bioactivities or generating toxic reaction products [6]. Third, many of them have a low oral bioavailability due to their poor solubility, stability, and absorption within the gastrointestinal tract. These challenges can often be overcome using encapsulation technologies. These technologies typically involve trapping the bioactive substance inside a carrier material, such as a small particle, capsule, or fiber. Encapsulation can increase the water dispersibility, wettability, chemical stability, and bioavailability of bioactive substances [7]. Moreover, it can control their release in different regions of the gastrointestinal tract, such as burst or prolonged release in the mouth, stomach, small intestine, or colon. For this reason, there is considerable interest in designing edible carriers that can improve the efficacy of bioactive agents [8].

Microcapsules produced by spray drying bioactive agents in the presence of suitable wall materials, such as starches, proteins, or gums, have been used to protect bioactives [9,10]. However, the size of microcapsules ranged from 5 μm to 200 μm. Microcapsules are less efficient at encapsulating active molecules and are difficult to be taken up by cells, with a poor delivery efficiency. However, nanocarriers (from 10 nm to 1000 nm) are conducive to improving the stability and solubility of the encapsulated active molecules, which can regulate the rate of release, increase the permeability of biological membranes, change their distribution in the body, and improve bioavailability [11]. For this reason, there has been great interest in the development of nanocarriers that have much smaller particle sizes and larger specific surface areas. Nanocarriers can be produced from food-grade ingredients, including lipids, phospholipids, proteins, and/or polysaccharides. In this article, we focus on the utilization of whey proteins for this purpose. Whey protein products, covered in the U.S. Food and Drug Administration’s (FDA) U.S. FDA Evaluation of Food Additives Safety Indicators (Generally Recognized as Safe (GRAS)) Bulletin, include colostrum whey proteins, whey isolate proteins, and natural whey proteins. Whey proteins are generally regarded as safe (GRAS) food ingredients and have a diverse range of functional attributes that are useful for assembling nanocarriers, including emulsifying, gelling, structuring, and binding properties [11]. Among the various proteins, whey protein has the highest nutritional value. Whey protein is a high-quality complete protein, also known as an animal protein. It contains eight kinds of essential amino acids, and the ratio is reasonable, close to the proportion of human needs for human body growth, development, anti-aging, and other life activities. Whey protein is easier to be digested and absorbed, and is a very good immunity-enhancing protein. Whey protein has a low content of fat and lactose, and has many health functions that are beneficial to the human body. It is a good choice of protein intake for infants and young children, the elderly, the post-operative population, and sports people. Whey proteins exhibit a lower allergenicity compared to soy proteins, peanut proteins, etc. The literature has reported that whey protein allergy mainly occurs in early infancy and reacts in all systems of the body, and clinically, it is mostly characterized by gastrointestinal and skin symptoms, and in severe cases, it can lead to shock and death [12]. Currently, whey proteins can be modified by physical, chemical, and biological methods to significantly reduce the allergenicity of whey proteins by changing the linear epitope or spatial conformation of the protein.

**Figure 2:**
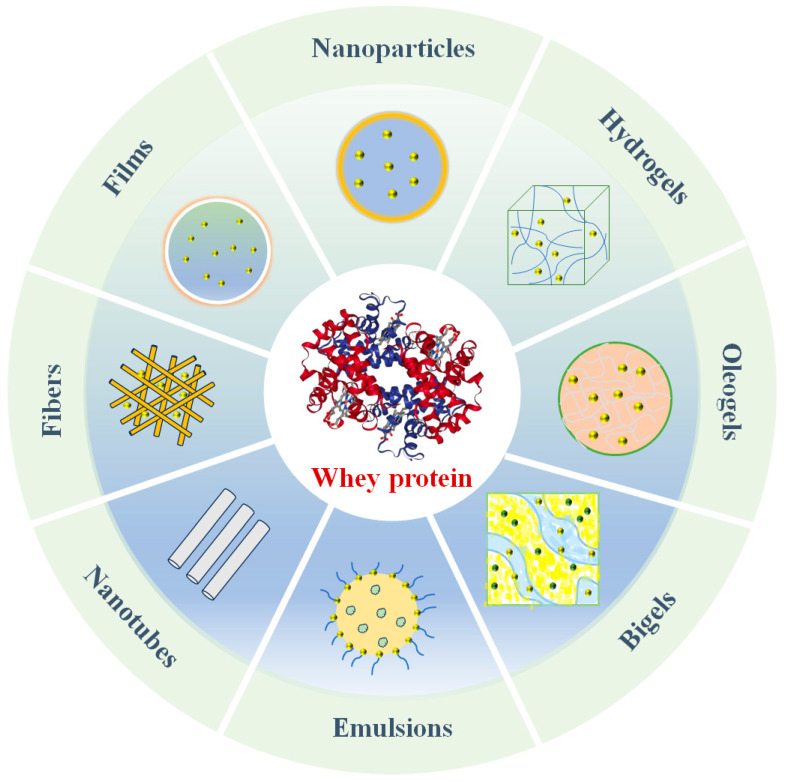
Different carrier forms for whey protein-based delivery systems.

Indeed, whey proteins can be used to assemble a diverse range of nanoscale carriers, including nanoparticles, hydrogels, oleogels, bigels, films, fibers, nanotubes, and nanoemulsions (Figure 2) [11]. Each kind of carrier has its own characteristics, which make them suitable for different applications in foods and other products. For example, nanoparticles are suitable for the development of powdered products, emulsions for oral liquid products, and gels for fat replacement or 3D-printed products. Moreover, they differ in their ease of preparation, cost, and robustness, which also impacts their application in commercial products. Consequently, it is important to identify the most suitable carrier for specific applications. Whey proteins exhibit various physiological functions beneficial to human health due to their unique structure (Table 1), for example, antibacterial, anticancer, anti-diabetes, antioxidant, antiviral, hypotensive, liver protection, the treatment of malnutrition and sarcopenia, and so on. Therefore, whey proteins have been widely studied for their use as carriers of active molecules, on the one hand, and for their ability to exert their own health activities, on the other.

Whey protein-based carriers are typically degraded in the human gastrointestinal tract by proteases and other digestive enzymes, which means they can be designed to deliver bioactive agents to specific regions by controlling their digestibility. In addition, whey proteins themselves are highly nutritious food ingredients, being a good source of essential amino acids [11,13]. For these reasons, whey protein-based carriers are being increasingly used by food researchers to create foods designed to improve human health and nutrition. Whey proteins can be prepared into a diverse range of carriers because they have diverse surface properties (such as hydrophobicity, charge, and chemical reactivity), which can be modulated by simple processing operations, like changing the pH, ionic strength, and temperature. As a result, the attractive and repulsive interactions between the proteins can be controlled to make them assemble into different structures, like spheres, sheets, or fibers.

**Table 1:** Health-promoting effects and mechanism action of whey proteins.

Health-Promoting Effect	Mechanism of Action			Refs.
Antibacterial	a. The antimicrobial impact is effective against bacterial pathogens and the rotavirus that leads to gastrointestinal infections	b. Various microorganisms, including Gram-negative bacteria, Gram-positive bacteria, yeast, filamentous bacteria, and parasitic protozoa, are effectively inhibited, along with certain antibiotic-resistant pathogens	c. Maximizing the intrinsic immune cell reaction by boosting phagocytosis	[14]
Anticancer	a. Great substitute for oral nutritional supplements for cancer patients due to its high-quality proteins, minimal lactose/fat content, and good digestion	b. Enhancing the immune system and nutritional health of cancer patients undergoing chemotherapy	c. Boosting glutathione levels in pertinent organs and activating the glutathione pathway to promote protection against cancer cells	[15]
Anti-diabetes	a. Stimulating insulin secretion	b. Significantly higher ability to stimulate insulin production compared to casein, milk, and numerous other proteins	c. Decreasing postprandial hyperglycemia in a dose-dependent manner	[16]
Antioxidant	a. Scavenging reactive oxygen species	b. Combining with ions of transition metals and postponing the peroxidation process	c. Increased antioxidant capacity through Maillard coupling	[17]
Antiviral	a. Binding to the respiratory syncytial virus and influenza virus in humans	b. Inhibiting virus replication	c. Maximizing the intrinsic immune cell reaction by boosting phagocytosis	[18]
Hypotensive	a. The ACE inhibitory method is used to reduce blood pressure	_	_	[19]
Liver protection	a. Enhancing liver function and elevating the level of plasma glutathione	b. Reducing the concentrations of triglycerides and cholesterol within the liver	_	[20]
Treatment of malnutrition and sarcopenia	a. Being a leucine-rich protein	b. Enhance muscle mass, muscle strength, and lower-extremity function in older individuals who have sarcopenia	c. Enhancing muscle protein synthesis after meals in older individuals with sarcopenia	[21]

Several examples of recent articles have reviewed research advances of protein-based delivery systems. These studies have focused on ferritin/defatted ferritin cages, plant-derived viral capsids, small heat-shock protein cages, albumin, collagen and gelatin, milk proteins, and soy proteins [13,22,23]. However, few articles have focused on the utilization of whey protein-based carriers for this purpose. In this paper, we therefore review recent advances in the use of whey proteins to construct carriers for bioactive agents. The fabrication, structure, functionality, and application of the various kinds of carriers that can be produced from whey proteins are discussed. In addition, the role of complexing or conjugating whey proteins with other food ingredients (such as polysaccharides, lipids, and different proteins) in improving their functional performance is highlighted.

Overall, our review is expected to provide new insights into the design, production, and application of whey protein-based carriers for enhancing the efficacy of bioactive agents in food and health. In addition, the different types of carriers are grouped and summarized in this study, and the advantages and disadvantages of different whey protein-based carriers were discussed. Notably, we further elaborate on the mechanism and advantages of and improvement in whey proteins as carriers from the perspective of their interaction with other substances, which is significantly innovative.

## 2. Overview of Whey Proteins

Whey protein is a by-product of the cheese production. The term “whey protein” actually refers to a mixture of proteins that are present in the supernatant (“whey”) that is produced when the pH of cow’s milk is lowered to 4.6 and the casein precipitates (“curd”). Whey protein consists of β-lactoglobulin, α-lactalbumin, bovine serum albumin, and various immunoglobulins. Depending on their protein content, commercial whey protein ingredients can be classified into whey protein concentrate (WPC), with a protein content of 50–85%, and whey protein isolate (WPI), with a protein content > 90% [24,25]. Several methods can be used to isolate whey proteins from cow’s milk, including salting out, isoelectric precipitation, organic solvent extraction, organic solvent precipitation, double aqueous phase extraction, and inverse micellar extraction.

Whey proteins are relatively small amphiphilic molecules that have good water solubility over a wide pH range [26]. From a nutritional perspective, whey protein has several advantages, including high purity, high bioavailability, high utilization rate, and a balanced essential amino acid profile. Consequently, it is widely used as a high-quality protein source in supplements and foods [27].

The ability of whey protein to assemble into different kinds of carriers is mainly based on its surface properties, such as hydrophobicity, electrical charge, and chemical reactivity [28]. Moreover, these surface properties can be altered by changing the solution or environmental conditions, such as pH, ionic strength, and temperature, which allows the molecular interactions of whey protein molecules to be controlled. For instance, controlled heating can cause whey proteins to partially unfold, which exposes non-polar and sulfhydryl groups on their surfaces, thereby increasing their tendency to form hydrophobic and disulfide bonds. Controlling the pH alters both the net charge and charge distribution on the surfaces of whey proteins, thereby altering their electrostatic interactions with themselves and other molecules. Adding mineral ions can also alter the electrostatic interactions between whey proteins through ion binding, ion bridging, and electrostatic screening effects. Indeed, the unique surface chemistry of whey proteins, as well as the ability to be able to change it through simple processing operations, are responsible for many of the functional attributes that make them suitable for constructing bioactive carriers, including their surface activity, self-assembly, gelation, film formation, and binding properties [29,30].

Previous studies have shown that whey protein-based carriers can be used to encapsulate, protect, and deliver a broad range of bioactive substances, including polyphenols, essential oils, nutraceuticals, vitamins, minerals, omega-3 fatty acids, bioactive peptides, and probiotics. For instance, in the food industry, researchers have encapsulated citrus peel extracts in whey protein nanoparticles, which slowed down their release under gastrointestinal conditions and enhanced their antioxidant activity [31]. In the pharmaceutical industry, whey protein-based nanoparticles have been used to encapsulate lycopene, which increased its bioavailability and anticancer effects [32]. They have also been used to increase the bioavailability of hydrophobic drugs (lopinavir and fenretinide) and to enhance the viability of probiotics [33]. These few examples highlight the potential of whey protein-based carriers for application in the food and pharmaceutical industries. More examples will be provided later when specific kinds of carriers are considered.

## 3. Whey Protein–Dietary Ingredient Interactions

The development of whey protein-based carriers requires knowledge of the interactions of whey proteins with other ingredients. It has been reported that proteins, polysaccharides, lipids, polyphenols, and metal ions can all interact with whey proteins and contribute to the enhancement in their functional properties and biological activities [34,35]. For example, the interaction of whey protein with quercetin has been reported to improve the former’s foaming, emulsifying, and antioxidant properties [36]. Similarly, the interaction of whey protein with soy isoflavones improved its solubility, foaming, and gelling properties [37]. In this section, we therefore review the interactions between whey proteins and other food ingredients.

### 3.1. Whey Protein–Polymer Interactions

Whey proteins may interact with polymers in foods, which are usually naturally molecules like proteins and polysaccharides, through physical or covalent interactions. The structural organization of the resulting mixed system can be classified into three main types: aggregation, phase separation, and mutual solubilization [38]. The nature of the system formed depends on environmental conditions (such as temperature, pH, and ionic strength) and polymer properties (such as concentration, molecular weight, charge, and conformation). The main molecular interactions involved in protein–polymer interactions are hydrogen bonding, electrostatic interactions, hydrophobic interactions, van der Waals forces, and covalent bonding. Information about the types of interactions involved can be obtained using a variety of methods, including solubility in different solvents, fluorescence spectroscopy, circular dichroism, isothermal titration calorimetry, dissipative quartz crystal microbalance, and molecular docking analysis [39].

If there are no strong attractive interactions between two polymers, they tend to form a uniform molecular solution in dilute systems [40]. However, if two polymers carry the same net charge (both positive or both negative), they may separate into two phases due to electrostatic repulsion when their concentration is sufficiently high. One of the phases is rich in one of the polymers but depleted in the other polymer, while the opposite is true for the other phase. Conversely, if two polymers carry opposite charges (one negative and one positive), they tend to associate with each other through electrostatic attraction and form coacervates or precipitates.

The electrical charge on whey proteins develops from positive to negative when the pH is raised from below to above their isoelectric point, which plays an important role in determining their electrostatic interactions. For instance, proteins may be repelled from anionic polysaccharides at pH values above their isoelectric point but attracted at lower pH values [41]. Moreover, two proteins with different isoelectric points may repel each other at some pH values (above or below both isoelectric points) but attract each other at other pH values (between the two isoelectric points). It should be noted, however, that whey proteins have both negative and positive charges on their surfaces at their isoelectric point, which means they can still participate in electrostatic interactions. Whey proteins also have numerous non-polar amino acids on their surfaces, when their fractions are partially denatured and more hydrophobic regions are exposed. This may lead to the formation of strong hydrophobic bonds with other polymers, such as proteins and polysaccharides with exposed non-polar groups. The hydrophobic forces between the hydrophobic amino acid residues of zein and whey proteins have been shown in studies to be the main driving force for the formation of zein–whey protein complexes [42,43].

The formation of conjugates (covalent links) or complexes (physical links) of whey proteins with other polymers often leads to an improvement in their functionality. For instance, the conjugation or complexation of whey proteins with polymers may mask protease hydrolysis sites, thereby inhibiting their digestion within the gastrointestinal tract, which can lead to the design of controlled delivery systems.

Proteins are commonly conjugated or complexed with polysaccharides to improve their performance [44]. Polysaccharides may be isolated from plant, animal, and microbial sources and exhibit a variety of functional properties and biological activities depending on their compositions and structures. Whey protein–polysaccharide complexes have been used for a wide variety of applications in foods and medicines, including as bioactive carriers, edible inks, fat substitutes, gels, and tissue scaffolds [45]. For instance, combining whey proteins with dandelion root polysaccharides improved the textural attributes and water-holding capacity of the composite gels formed [46]. Combining whey proteins with sweet tea polysaccharides was also shown to improve the texture and water-holding capacity of the composite hydrogels [47]. Mixing whey proteins with either guar gum, xanthan gum, acacia bean gum, or gum arabic was reported to improve their rheological properties, which facilitated their application as edible inks in 3D food printing [48].

### 3.2. Whey Protein–Small Molecule Interactions

The functional performance of whey proteins can also be enhanced by combining them with small molecules [26]. The interactions of small molecules with whey proteins depend on the number and affinity of binding sites on the protein surfaces. Binding constants (*k*) are commonly used to quantify the binding affinity between whey proteins and small molecules [49]. The binding properties of proteins can be conveniently assessed using fluorescence quenching methods, which are based on measuring changes in the fluorescence intensity due to interactions of small molecules with protein surfaces, especially phenolic groups [50].

Many small molecules found in foods exhibit health-promoting functions, such as antioxidant, anti-inflammatory, anticancer, and anti-atherosclerotic effects [6]. As mentioned earlier, however, many of these bioactive molecules have a low water solubility and poor stability when exposed to heat, oxygen, light, and alkaline conditions, thereby limiting their use in functional foods. Whey protein-based carriers can be designed to improve the water dispersibility, stability, and bioavailability of bioactive molecules. This application of whey proteins has been discussed in detail in several review articles and so will not be repeated in this paper [51]. However, it is worth pointing out that the design and development of these whey protein-based carriers have contributed significantly to enhancing the efficacy of many bioactive molecules.

Whey protein–small molecule interactions affect the structural properties of whey proteins. Depending on the nature of the interaction, this effect may lead to a range from minor to major structural changes. The binding of some bioactive molecules to whey proteins causes a decrease in their α-helix or β-folding contents and an increase in their random coil content, which leads to a whey protein structure that is more open and flexible [52]. Conversely, some bioactive molecules have the opposite effect, with a little or no effect on protein structure. Changes in the structure of whey proteins often lead to changes in the functional properties. In some cases, interactions between whey proteins and small molecules increases the solubility of the whey proteins, which has been attributed to their ability to bind to non-polar regions on the protein, thereby reducing their surface hydrophobicity. The foaming properties of whey proteins can also be enhanced by regulating their interactions with small molecules [50]. The interaction of whey proteins with small molecules can protect them from degradation by pepsin in the human gastrointestinal tract, as well as reducing their allergenicity [53]. In summary, small molecules can either increase or decrease the functional performance of whey proteins depending on the nature of the interactions. Therefore, it is crucial to investigate the interactions between different types of small molecules and whey proteins and to elucidate their interaction mechanisms when selecting a protein for a particular application.

### 3.3. Whey Protein–Other Dietary Ingredient Interactions

Whey proteins can also interact with lipids in various ways, which can promote their structure and functional attributes. The nature of the interactions is strongly influenced by the lipid type, e.g., triacylglycerols, diacylglycerols monoacylglycerols, free fatty acids, phospholipids, or surfactants. For example, non-ionic surfactants may bind to non-polar patches on the surfaces of whey proteins through hydrophobic attraction, which reduces the surface hydrophobicity of the protein [54]. Anionic surfactants can interact with whey proteins through a combination of electrostatic and hydrophobic attraction, which can promote protein unfolding and reduce protein aggregation [55]. Whey proteins can also form complexes with phospholipids and free fatty acids through a combination of electrostatic, hydrogen bonding, and hydrophobic interactions [56,57]. Typically, the non-polar tail group of anionic polar lipids interacts with non-polar groups on the protein surfaces, whereas the anionic polar head groups interreact with cationic and polar groups on the protein surfaces. These interactions may alter the behavior of the whey proteins in the gastrointestinal tract, such as their interactions with mixed micelles or cell membranes. Moreover, these interactions may improve the emulsifying properties of whey proteins, which makes them more suitable for encapsulating bioactive lipids in emulsions or nanoemulsions [42]. When whey proteins adsorb to oil–water interfaces (which are often composed of triacylglycerol molecules), they tend to unfold due to alterations in their molecular environment [58]. This conformational change exposes reactive groups at the protein surfaces, such as non-polar and sulfur-containing amino acids, which can promote interfacial crosslinking, which has been shown to alter the stability and performance of whey protein-coated lipid droplets [54,59]. Studies on whey protein–lipid interactions may therefore provide useful information when designing carriers for bioactive agents [60].

Whey proteins have also been shown to interact with various kinds of vitamins, which can enhance the dispersibility, stability, or bioavailability of the vitamins. For example, the water dispersibility and chemical stability of vitamin E was significantly enhanced when it was combined with whey protein [61]. In another study, the water dispersibility and oxidative stability of vitamin D_3_ was improved by encapsulating it within whey protein nanoparticles. Whey protein–vitamin interactions can therefore improve the functional performance of vitamins. However, they can also alter the structure and functionality of whey proteins. For example, the interaction of vitamin A with bovine serum albumin enhanced the structural stability of the protein [62]. However, the interaction between β-lactoglobulin and vitamin B_12_ had little effect on the structure of the protein [63]. Thus, the effects of protein–vitamin interactions depend on the molecular and physicochemical characteristics of the vitamins.

The interactions of whey proteins with mineral ions may affect their structure, interactions, and functionality. Mineral ions vary in their charge (anionic or cationic), valence (1, 2, 3…), and dimensions (small to large), for example, K^+^, Na^+^, Ca^2+^, Cl^−^, and SO_4_^2−^, which impacts their effects on whey proteins. Mineral ions can alter the surface properties of whey proteins by binding to oppositely charged groups on their surfaces. For instance, Ca^2+^ ions can bind to carboxyl groups (-CO_2_^−^) on the surfaces of proteins. Moreover, they can alter the interactions between protein molecules by forming salt bridges or screening electrostatic interactions [64].

An understanding of the interactions between proteins and other food ingredients is therefore critical when developing whey protein-based carriers for bioactive agents.

## 4. Types of Whey Protein-Based Carriers

**Figure 3:**
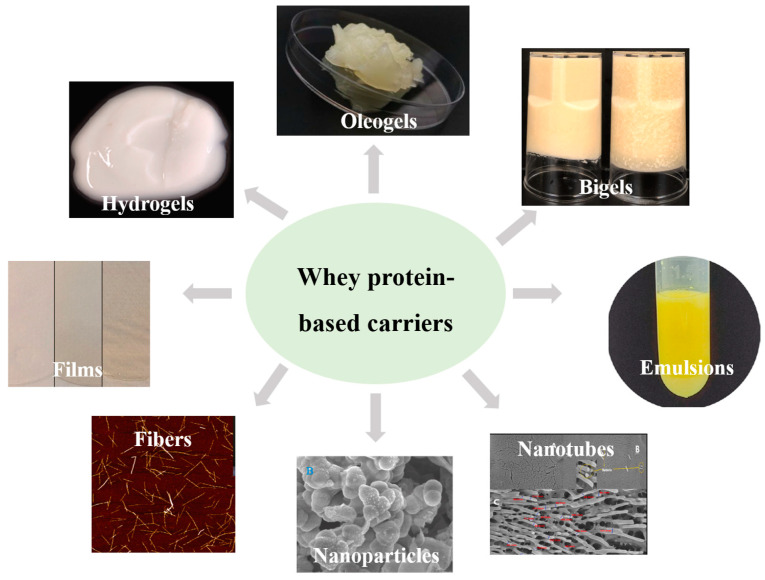
Morphological characteristics of different whey protein-based carriers [65,66,67,68,69,70,71,72].

In this section, different kinds of bioactive carriers that can be produced from whey proteins are discussed, including their morphology (Figure 3), preparation, structure, and properties.

### 4.1. Biopolymer Nanoparticles

Whey protein-based biopolymer nanoparticles have been widely investigated for their potential as carriers of bioactive agents in the food and medical industries. These carriers consist of relatively small (<1000 nm) spherical particles that are mainly composed of crosslinked whey protein molecules and sometimes other biopolymers. Food matrices contain charged particles that interact with each other and with the medium and are generated through several interfacial processes and mechanisms. The zeta potential is one of the most useful parameters for studying electrical interactions in food systems. The size and zeta potential of biopolymer nanoparticles can be measured by dynamic light scattering [73].

Whey protein nanoparticles can be fabricated using a variety of methods, including antisolvent precipitation, directed self-assembly, and electrospraying. Bioactive propolis extracts have been encapsulated within whey protein nanoparticles, which improved their water dispersibility and stability [74]. Soy isoflavones have been encapsulated in whey protein nanoparticles using a pH-driven approach, which enhanced their stability and bioaccessibility. Olive leaf phenolics have been encapsulated in whey protein nanoparticles using the electrospraying method [75]. The protein nanoparticles produced were relatively small (230–660 nm), had a high encapsulation efficiency, and enhanced the water dispersibility of the phenolics. Composite biopolymer nanoparticles comprising whey proteins and other biopolymers can also be used to encapsulate bioactive agents [76]. These other biopolymers may be polysaccharides or different kinds of proteins, which alter the nature of the nanoparticles formed, as well as their functional performance. Table 2 summarizes studies of whey protein-based biopolymer nanoparticles used for the encapsulation, protection, and delivery of bioactive agents. Whey protein-based nanoparticles can be prepared by antisolvent precipitation, self-assembly, pH-induction, and electrospray. They can make individual whey protein nanoparticles, or whey protein–protein composite nanoparticles, and whey protein–polysaccharide composite nanoparticles, with average sizes ranging from 58 nm to 600 nm, which exhibit good encapsulation efficiencies for biologically active molecules and have a great potential for application in the encapsulation and protection of LSAMs. Overall, whey protein-based nanoparticles are biocompatible and can be used to enhance the bioavailability of bioactive molecules and nutrients by encapsulating them and enabling controlled and targeted in vivo release. However, nanoparticles are susceptible to environmental factors, such as pH and temperature, and are less stable. Moreover, the preparation of nanoparticles requires high processing conditions and equipment, and the above issues need to be addressed before large-scale production.

### 4.2. Hydrogels, Oleogels, and Bigels

Gels are semisolid materials that consist of a 3D network of polymers or particles that entraps a fluid. Different kinds of edible gels can be formed depending on the nature of the fluid phase: hydrogels (water), oleogels (oil), and bigels (mixture). There are various methods of preparing gels depending on the polymers or particles that make up the network structure, such as coagulation, evaporation, and crosslinking [86]. Gels can also be prepared with macroscopic or microscopic dimensions depending on the preparation method used. For instance, microgels can be produced by injecting a mixture of proteins into a suitable crosslinking solution.

Hydrogels contain a 3D network of crosslinked polymers or particles, which entrap an aqueous fluid phase through hydration and capillary forces [87]. The networks in whey protein-based hydrogels may be held together by covalent or non-covalent bonds, such as hydrogen bonding, hydrophobic forces, electrostatic interactions, and/or van der Waals forces. These kinds of hydrogels have been used to encapsulate, protect, and deliver various types of bioactives. For example, Gun’ko et al. prepared whey protein-based hydrogel beads and explored the encapsulation properties of riboflavin in them and the release pattern under gastrointestinal simulated digestion. After a drying treatment at 30 °C, the hydrogel beads underwent swelling upon entering the gastrointestinal environment and released large amounts of riboflavin within one hour in the gastric (58%) and intestinal (34%) environments. The rate of riboflavin release was significantly reduced after hydrogel drying compared to before drying (rate constant Kr: 0.1 decreased to 0.016), suggesting that, after drying, the whey protein hydrogel beads have the potential to act as an orally functionalized factor and deliver it to the intestinal tract [88]. In another study, whey protein–carrageenan composite hydrogels were used for curcumin loading. The results showed that the physical stability of curcumin was significantly improved by the composite hydrogel loading, and the composite hydrogel had a higher curcumin encapsulation capacity than the whey protein gel alone. In addition, the composite hydrogel (87%) could achieve an efficient colonic delivery of curcumin in simulated digestion experiments, which was much higher than the delivery efficiency of whey protein gel alone (31%) for curcumin. Whey protein-based hydrogel systems are expected to be an efficient encapsulation and delivery vehicle for bioactive molecules such as curcumin [89]. Whey protein-based hydrogels have excellent hydration properties that favor the retention of moisture in food products. The natural degradation properties make them environmentally friendly and can be utilized in a variety of high-moisture food systems. However, whey protein-based hydrogels are mechanically weak and sensitive to pH and temperature, and future research needs to focus on developing whey protein-based hydrogels with good mechanical properties and stability to better meet the needs of different food systems.

Oleogels consist of a 3D network of an oleogelator (which is typically a polymer or particle) that traps an oily phase. Unlike polymers used for hydrogels, oleogels utilize small amphiphilic molecules that self-assemble through highly specific non-covalent interactions and entrap liquid oils by capillary forces. Oleogels have made great strides in mimicking the desired sensory properties while maintaining the healthy nutrient content of the oil [90]. Protein-based oleogels can be formed using a variety of methods, including emulsion-, foam-, and hydrogel-templated methods [91]. Whey protein-based oleogels have been developed to encapsulate fish oil, which was shown to improve its oxidative stability. The incorporation of polyphenols into whey protein-based oleogels was shown to further improve the oxidative stability of polyunsaturated oils. Moreover, the presence of polyphenols in whey protein-based oleogels was reported to alter their digestibility and bioaccessibility. Whey protein-based oleogels have been developed to achieve fat replacement while encapsulating bioactive molecules for a synergistic effect with fat reduction and nutritional value. However, how to achieve the desired texture, consistency, and stability are still problems for its use as a fat substitute.

Bigels typically consist of an interpenetrating mixture of oleogels and hydrogels, which leads to novel optical, textural, and stability characteristics, as well as the ability to simultaneously encapsulate both hydrophilic and hydrophobic bioactives. Whey protein containing bigels have been explored for their potential application in the food and medical industries. For instance, bigels have been produced by blending oleogel emulsions (soy lecithin, stearic acid, soybean oil, and water) with hydrogels (whey protein and water). These bigels were shown to be predominantly solid-like over a wide range of temperature, and their textural attributes could be controlled by varying the oleogel-to-hydrogel ratio. Similarly, bigels have been produced by blending an oleogel (aggregated whey protein in sunflower oil) and hydrogel (heated whey protein solution) together. Whey protein-based bigels have been used to successful encapsulate and protect probiotics during simulated gastrointestinal digestion [42]. Whey protein-based bigels combine the properties of both hydrogels and oleogels with excellent structural and functional properties. However, complex formulations and the stability of the oil–water interface are still challenges in the development and application of bigels.

Taken together, these studies show that whey proteins can be used to create a variety of different gelling systems, for example, hydrogels, oleogels, and bigels, which may be useful as carriers of different kinds of bioactive agents, but more research, such as animal modeling and safety assessment, is still needed.

### 4.3. Films, Fibers, and Nanotubes

Whey proteins can also be used to create films, fibers, and nanotubes due to their ability to interact with each other through a variety of different ways depending on environmental and solution conditions. Whey protein-based films can be fabricated by methods equivalent to those used to manufacture conventional polymer-based films, which include casting, compression, and even extrusion. Films prepared from pure whey protein are typically rigid and brittle with low flexibility and poor barrier properties. In order to improve the mechanical properties of whey protein-based films, other bio-based materials are usually added for compounding. Whey proteins can be used to create edible films or coatings that may replace petroleum-based ones in some applications, thereby reducing the environmental damage caused during the production and disposal of plastics [28]. As an example, whey protein-based films containing soybean extracts have been prepared that exhibited good transparency, antioxidant activity, and antimicrobial activity [92]. In another study, composite films containing whey proteins, cellulose nanocrystals, and essential oils were shown to exhibit good antioxidant and antimicrobial properties, which may be useful for their application as food packaging materials for meat, seafood, fruits, and vegetables. Among them, the whey protein composite films showed 77%, 75%, and 80% increases in antimicrobial activity against Staphylococcus aureus, Lactobacillus monocytogenes, and S. enteritidis, respectively. This study offers great potential for the development of sustainable multifunctional antimicrobial packaging materials [93]. Above all, whey protein films are characterized by biodegradability, palatability, and good gas and oil barrier properties. However, difficulties such as mechanical strength, water sensitivity, and processing complexity remain challenges for their industrial application.

Whey proteins have also been used to create fibers that can be used to encapsulate, protect, and control the release of bioactives. The diameters of the zones of significant inhibition of whey protein fibers against S. aureus were 20.0 ± 0.8 mm, 22.5 ± 0.5 mm, and 35.5 ± 0.6 mm, respectively, while the diameters of the zones of inhibition against E. coli were 11.5 ± 0.8 mm, 16.6 ± 0.7 mm, and 23.8 ± 0.5 mm for ATCC 25922 [94]. Nanofibers created from whey proteins using electrospinning methods can be used to encapsulate and control the release of bioactives. Composite fibers have been produced by blending whey proteins with other substances, including soy proteins, chitosan, starch, and dextran [95,96,97]. Whey protein-based nanofibers have a high surface area to volume ratio, which facilitates the encapsulation and release of bioactive molecules and can be used for the development of delivery systems and scaffolding. However, the preparation of nanofibers requires specialized equipment, such as electrostatic spinning, and the cost of large-scale production is a challenge that hinders their development.

Some whey proteins can self-assemble into nanotubes under appropriate conditions, which can then be used for the encapsulation, protection, and controlled delivery of bioactives. Whey protein nanotubes consist of open cylinders of variable length with two open ends that can be used to trap bioactives inside and control their release profiles. These nanotubes can also be disassembled by altering the pH, which makes them suitable for pH-triggered release applications. Researchers have encapsulated caffeine within whey protein nanotubes as a means of controlling their release profiles [98]. They showed that a high encapsulation efficiency (about 100%) and loading capacity (about 10%) could be achieved, and that the caffeine-loaded nanotubes had a good resistance to freeze-drying. Other researchers have encapsulated curcumin within whey protein–polysaccharide nanotubes, such as α-lactalbumin–chitosan and bovine serum albumin–κ-carrageenan ones [99]. They reported that the encapsulation efficiency of curcumin was in the range of 40–45% and that the curcumin-loaded nanotubes exhibited some anticancer effects using a cell culture model. Whey protein-based nanotubes have a high specific surface area and show excellent performance in the adsorption and encapsulation of bioactive molecules. The same challenge of high preparation cost is faced by nanofibers.

### 4.4. Emulsions

The emulsifying properties of whey proteins means then can be used in the development of emulsion-based delivery systems for bioactives. In particular, they can be used to form oil-in-water emulsions or nanoemulsions by forming a protective coating around the oil droplets. For example, whey protein-stabilized oil-in-water emulsions have been used to encapsulate β-carotene, which improved its water dispersibility and stability [100,101]. High internal-phase Pickering emulsions (HIPPEs) stabilized by whey protein particles have also been used to encapsulate β-carotene, which improved its stability and bioaccessibility [102]. Similarly, whey-protein stabilized HIPPEs have been used to encapsulate and improve the bioaccessibility of lignocellulosic xanthophylls [103]. Lycopene has been successfully encapsulated within oil-in-water emulsions containing oil droplets coated by whey protein and chitosan, which improved its water dispersibility and chemical stability [104]. Other emulsions containing oil droplets coated by whey protein–polysaccharide layers have also been used to encapsulate and protect β-carotene [105,106]. Pickering emulsions containing oil droplets coated by colloidal whey protein particles have also been used to encapsulate a variety of bioactives. For instance, they have been successfully used to encapsulate and protect curcumin [107,108]. These studies show that whey proteins can either be used as molecular or particle emulsifiers to form oil-in-water emulsions or nanoemulsions that can be used to encapsulate, protect, and deliver hydrophobic bioactives. Overall, whey protein-based emulsions are suitable as delivery vehicles for hydrophobic bioactive molecules with good stability and shelf-life and are widely used in food, pharmaceutical, and cosmetic industries. However, the preparation process requires high-energy methods, such as high-pressure homogenization or ultrasonication.

## 5. Conclusions and Future Prospects

This review has shown that whey proteins are highly versatile molecules that can be used to construct a wide variety of different carriers for bioactive agents, such as vitamins, minerals, phytochemicals, and probiotics. The encapsulation of these substances in whey protein-based carriers can improve their dispersibility in water, their chemical stability during food processing, distribution, and storage, and their bioavailability after ingestion. Nevertheless, because there is a broad spectrum of whey protein-based carriers available, it is important to select the most appropriate one for the specific application. This choice will be based on a variety of considerations, including the nature of the bioactive (such as polarity, size, and stability), as well as practical considerations such as the cost, simplicity, scalability, and repeatability of the carrier fabrication process. In future, more research is required to compare the efficacy of different kinds of whey protein-based carriers for different kinds of bioactives under standardized conditions, so that the most appropriate ones can be identified.

Future development of whey protein-based carriers for the delivery of bioactive molecules will benefit from advances in technology, sustainability, personalized nutrition, and regulatory frameworks. Addressing these issues is critical to achieving the full potential of these systems and gaining widespread consumer acceptance. First, develop more precise and efficient nanotechnology methods to achieve a uniform particle size and improved bioavailability of the encapsulated compounds. Explore hybrid systems that combine whey proteins with other nanomaterials to enhance functionality. Utilize microfluidics to control the production of whey protein nanoparticles of uniform size and consistent properties. Investigate compatibility and encapsulation efficiencies for a broader range of bioactive molecules, including peptides, polyphenols, probiotics, and essential oils. Tailor encapsulation techniques to optimize the protection and release of these different bioactives. Develop biodegradable delivery systems to minimize post-consumer environmental impact. Innovate eco-friendly packaging solutions to complement the sustainable production of whey protein-based carriers. Tailor personalized delivery systems using genomics and metabolomics to meet individual nutritional and therapeutic needs. Develop customized packaging strategies to address specific dietary restrictions and health conditions. Integrate whey protein carriers into precision medicine frameworks to enhance targeted therapeutic delivery based on individual patient profiles. Stay abreast of developments in regulatory guidelines to ensure compliance with international standards for food safety and pharmaceutical applications. Collaborate with regulators early in the development process to facilitate smooth approval and market entry.

## Figures and Tables

**Table 2:** Whey protein-based nanoparticles for LSAM encapsulation and protection.

Carriers	Sample Preparation Method	Particle Size (nm)	Encapsulation Efficiency (%)	Guest Molecules	Potential Advantage	Refs.
whey protein nanoparticles	denatured by ethanol	58–126	_	_	particle size control	[77]
whey protein nanoparticles	nanofibrillated	78.20–270.44	63.84–84.33	propolis extract	controlled release in gastrointestinal digestion	[74]
zein–whey protein nanoparticles	pH-driven method	About 410	_	_	a better in vitro antioxidant activity	[78]
zein–whey protein composite nanoparticles	drop-by-drop anti-solvent precipitation method	152	About 69	cannabidiol	improved antioxidant activity and release of cannabidiol	[79]
whey protein isolate–phytosterol nanoparticles	anti-solvent method	215.9–253.8	81.55–95.40	phytosterols	a better emulsifying property	[80]
whey protein isolates and zein composite nanoparticles	pH-driven method	79.4–184	44.37–86.656	curcumin	Solubility of curcumin was enhanced	[81]
whey protein isolate/short linear glucan core–shell nanoparticles	self-assembly	70–150	Over 90% (Curcumin/WPI: 1:7, 1:6, 1:5, 1:4)	curcumin	improved sustained release and antioxidative activities	[82]
whey protein nanoparticles	electrospraying	232.3–659.8	36.66–83.66	olive leaf phenolics	encapsulation efficiency increased significantly at higher WPC levels	[75]
whey protein isolate–zein core–shell complex nanoparticles	pH-shifting method	90–150	_	fish oil	exhibited the excellent stability under environmental stresses	[83]
whey protein concentrate nanoparticles	calcium ion crosslinking	184–195.6	13.7–28.8	mandarin peel extracts	controlled the release of flavonoids and antioxidant was protected	[31]
whey protein nanospheres	electrospray	187 ± 2.71	87.93–94.47	bioactive compounds of Tinospora cordifolia	complete release of bioactive compounds responsible for anti-diabetic activity was observed after 24 h	[84]
whey protein isolate nanoparticles	high-speed agitator and ultrasonic assistance	243.20–269.03	_	curcumin	show preferable printability	[85]
whey protein isolate nanoparticles	single-step ethanol desolvation method	100–350	50.0–68.3	lycopene	signifying their feasible usefulness in cancer therapeutics and intervention	[32]

## Data Availability

Not applicable.
